# Supplementary material for: Development of a sequence-based in silico OspA typing method for Borrelia burgdorferi sensu lato
Source: Microb Genom. 2024 May 24;10(5):001252. doi: 10.1099/mgen.0.001252 (PMC11165634; doi:10.1099/mgen.0.001252)
Supplement: Uncited Supplementary Material 1. [file mgen-10-01252-s001.pdf]

SUPPLEMENTARY MATERIALS

Development of a sequence-based *in silico* OspA typing method for *Borrelia burgdorferi* sensu lato

Jonathan T. Lee [a], Zhenghui Li [a], Lorna D. Nunez [a], Daniel Katzel [b], B. Scott Perrin Jr. [b], Varun Raghuraman [a], Urvi Rajyaguru [a], Katrina E. Llamera [a], Lubomira Andrew [a], Annaliesa S. Anderson [a], Joppe W. Hovius [c], Paul A. Liberator [a], Raphael Simon [a], Li Hao [a]*

[a] Vaccine Research and Development, Pfizer Inc., Pearl River, NY, 10965

[b] Pfizer Digital, Pfizer Inc., Pearl River, NY, 10965

[c] Amsterdam University Medical Centers (UMC), location Academic Medical Center (AMC), Department of Internal Medicine, Division of Infectious Diseases, Center for Experimental and Molecular Medicine, University of Amsterdam, Amsterdam, Netherlands

Figure Legends

Figure S1. Comparison of OspA sequence identity for ISTs 13-17. Maximal sequence identity between variants belonging to each IST are shown in blue boxes. For variants from each IST, distribution of maximal sequence identity to variants not in the same IST are shown in red. Values for similarity threshold are depicted below the plot, calculated as the midpoint of the tails of the two distributions.

Figure S2. Alignment of IST consensus OspA sequences. Consensus sequences were determined based on all variants belonging to each IST. Single variants are instead depicted when 2 or less unique sequences comprise an IST.

Figure S3. Alignment of IST3 OspA sequences. Full-length OspA alignments of representative IST3 variants.

Figure S4. Simulated NGS coverage for a *B. burgdorferi* and *B. garinii* isolate. Short-read data were simulated using SimuSCoP v1.0 and aligned to *ospA* reference sequences for ISTs 1-6 to determine per-base coverage.

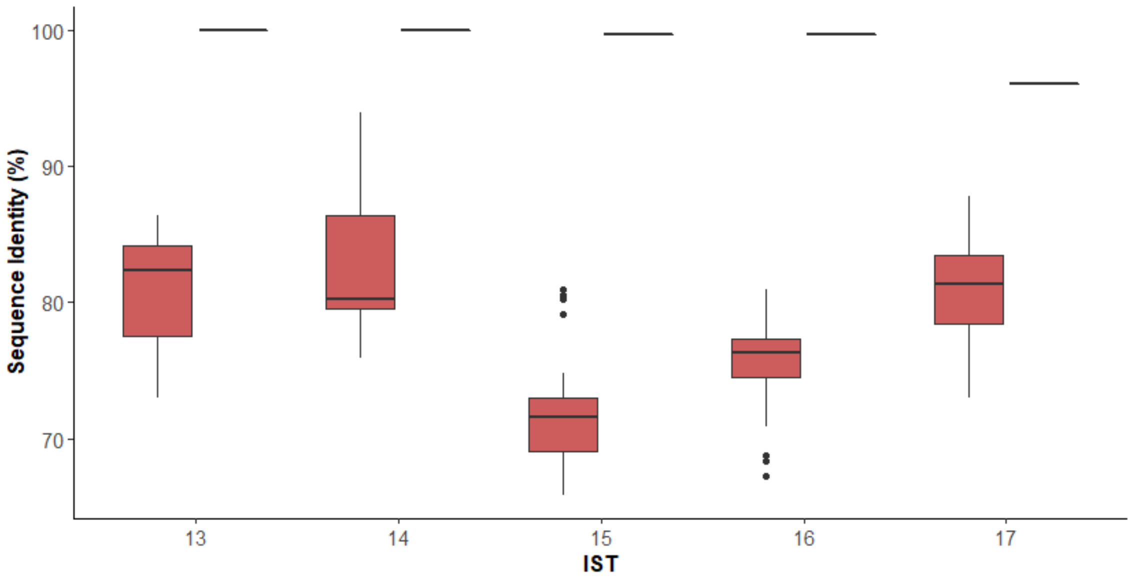

93.1	96.7	90.3	90.3	91.9

Figure S1. Comparison of OspA sequence identity for ISTs 13-17.

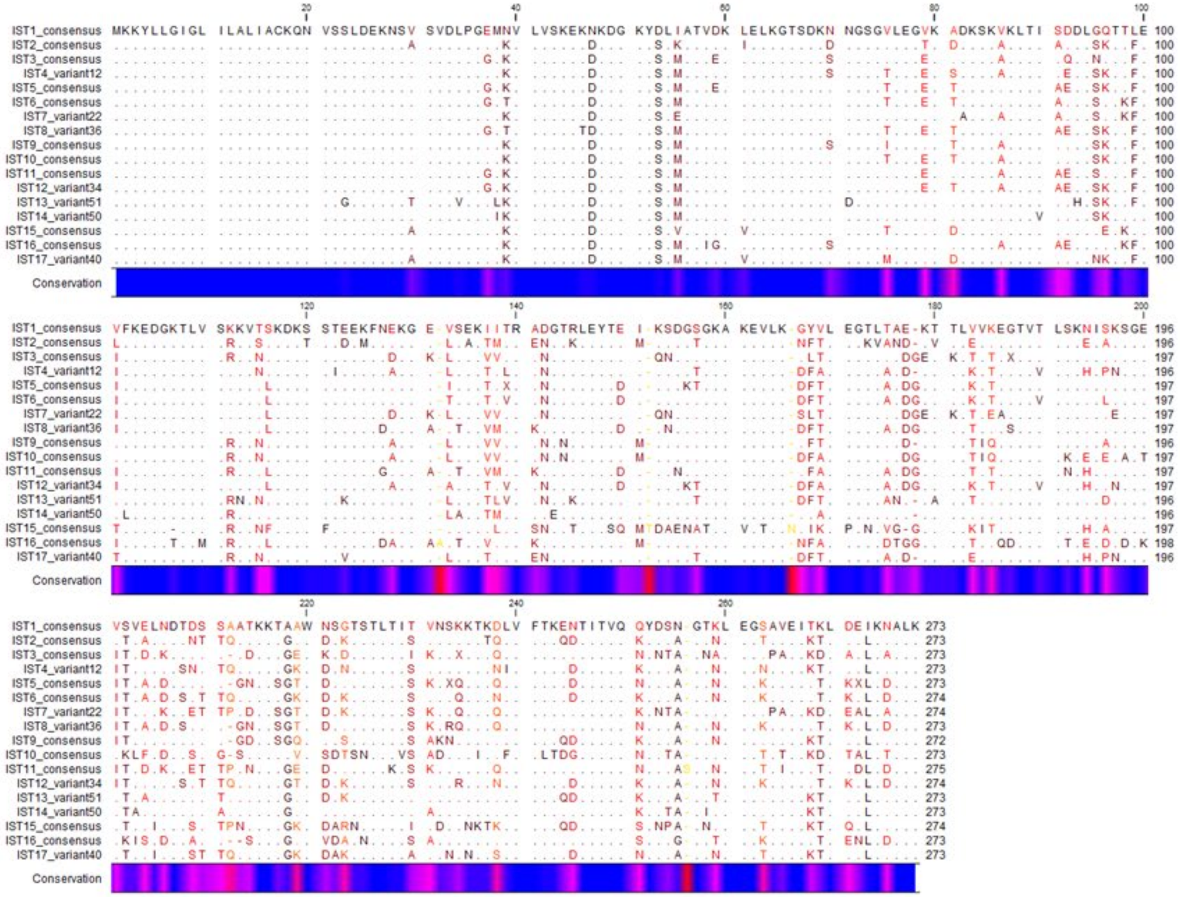

Figure S2. Alignment of IST consensus OspA sequences.

```
                         20         40         60         80
IST3 variant 10  MKKYLLGIGL ILALIACKQN VSSLDEKNSV SVDLPGGMKV LVSKEKDKDG KYSLMATVEK LELKGTSDKS NGSGVLEGEK 80
IST3 variant 11  .......... .......... .......... .......... .......... ........D. .......... .......... 80
IST3 variant 39  .......... .......... .......... .......... .......... .......... .......... .......... 80
 IST3 variant 9  .......... .......... .......... .......... .......... .......... .......... .......... 80
IST3 variant 38  .......... .......... .......... .......... .......... ........D. .......... .......... 80
IST3 variant 58  .......... .......... .......... .......... .......... ....E..... .......... .......... 80
IST3 variant 64  .......... .......... .......... .......... .......... .......... .......... .......... 80
IST3 variant 80  .......... .......... .......... .......... .......... .......... .......... .......... 80

                        100        120        140        160
IST3 variant 10  ADKSKAKLTI SQDLNQTTFE IFKEDGKTLV SRKVNSKDKS STEEKFNDKG KLSEKVVTRA NGTRLEYTGI QNDGSGKAKE 160
IST3 variant 11  .......... .......... .......... .......... .......... .........K D.......D. .......... 160
IST3 variant 39  T......... ..E....... .......... .......... .......... .......... ........E. .SN....... 160
 IST3 variant 9  .......... .......... .......... .......... .......... .......... ........E. K......... 160
IST3 variant 38  .......... .......... .......... .......... .......... .......... ........E. K.....N... 160
IST3 variant 58  .......... .......... .......... .......... .......... .......... D.......E. .......... 160
IST3 variant 64  .......... .......... .......... .......... .......... .......... ........D. .D....N... 160
IST3 variant 80  .......... .......... .......... .......... .......... .......... ........D. .D....N... 160

                        180        200        220        240
IST3 variant 10  VLKGLTLEGT LTADGETKLT VTEKTVTLSK NISKSGEITV DLKDTDSS-A DKKSGTWDSD TSTLTIIKNS LKTKQLVFTK 239
IST3 variant 11  ..A....... .......... .......... .......... ........-. .......... .......... K......... 239
IST3 variant 39  .......... ...G...... ...D...... S......... ........-V .......... .......... R......... 239
 IST3 variant 9  ....FA.... ..DG...... ...G...... .......... A.N..ETTP. ...T.E.K.. ......S... Q......... 240
IST3 variant 38  .......... .......... .......... .......... A.N..ETTP. ...T.E.K.. ......S... Q......... 240
IST3 variant 58  .......... ..N....... .E.G...... .......... .....ETTP. ...T.E.K.. ......S... R......... 240
IST3 variant 64  .......... .......... .E.G...... .......... T.G.....-. ...T.E.K.. .......... K....F.... 239
IST3 variant 80  .......... .......... .K.G...... .......... T.G.....-. ...T.E.K.. .......... K....F.... 239

                        260
IST3 variant 10  ENTITVQNYN RAGNALEGSP AEIKDLAELQ AALK 273
IST3 variant 11  .......... S......... D......K.. .... 273
IST3 variant 39  .......K.. T......... .........K .... 273
 IST3 variant 9  .......... .......... .........K .... 274
IST3 variant 38  .......... T..TK..... .........K .... 274
IST3 variant 58  .........D S..TK..... ......T..K T... 274
IST3 variant 64  .D.....K.. T..T....N. ......T..K T... 273
IST3 variant 80  .D.....K.. T..T....N. ......T..K T... 273
```

Figure S3. Alignment of IST3 OspA sequences.

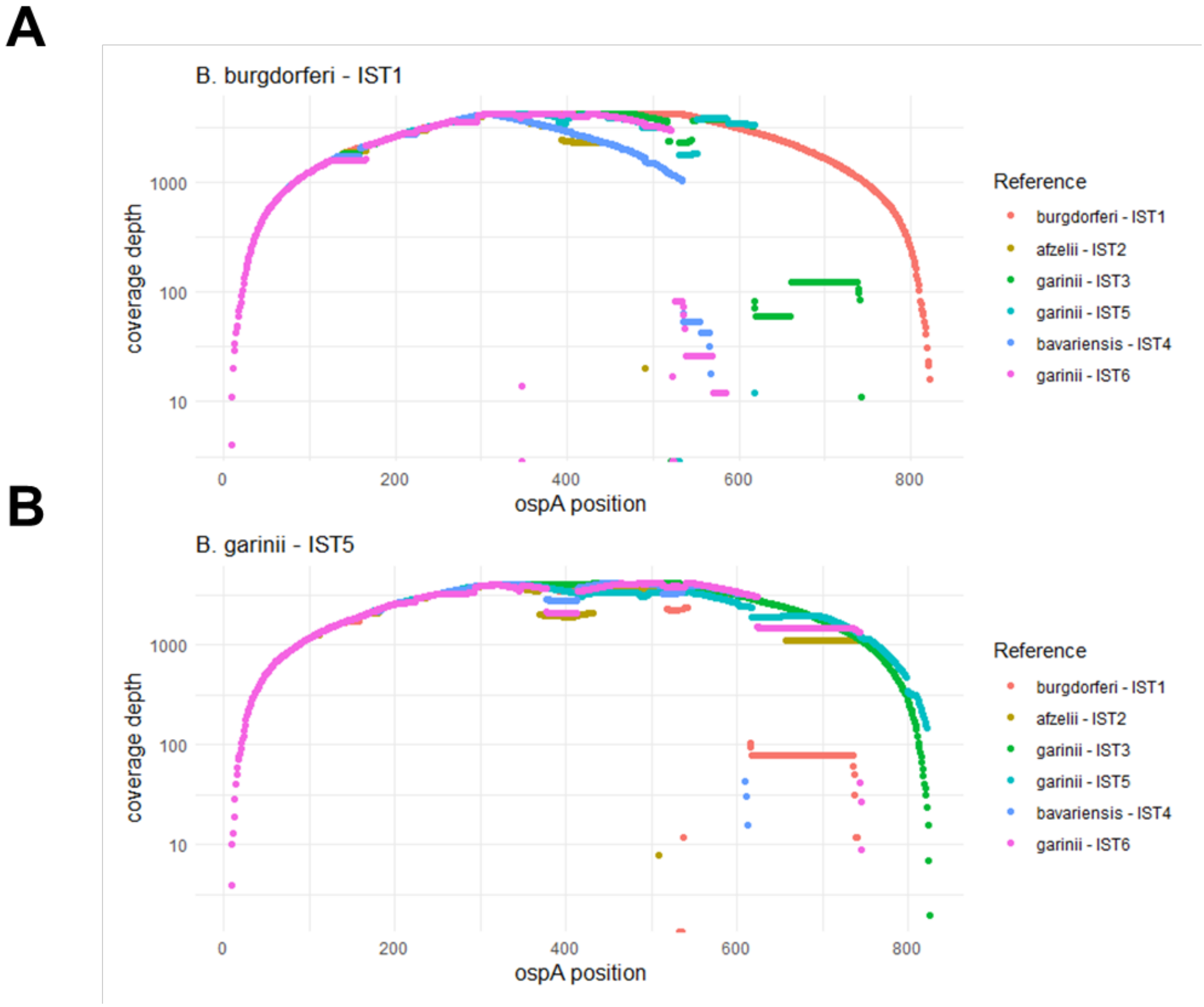

Figure S4. Simulated NGS coverage for a *B. burgdorferi* and *B. garinii* isolate.
